# Supplementary material for: Lactobacilli in COVID-19: A Systematic Review Based on Next-Generation Sequencing Studies
Source: Microorganisms. 2024 Jan 29;12(2):284. doi: 10.3390/microorganisms12020284 (PMC10891515; doi:10.3390/microorganisms12020284)
Supplement: Supplementary file 1 [file microorganisms-12-00284-s001.zip › Supplement S1.pdf]

Supplement S1

Search strategy to PubMed/MEDLINE:

(COVID-19[mh] OR COVID19[tw] OR 2019 Novel Coronavirus Disease[tw] OR 2019 Novel Coronavirus Infection[tw] OR COVID-19 Pandemic*[tw] OR COVID-19 Virus Disease[tw] OR COVID-19 Virus Infection[tw] OR Coronavirus Disease 2019[tw] OR Coronavirus Disease-19[tw] OR SARS Coronavirus 2 Infection[tw] OR SARS-CoV-2 Infection[tw] OR SARS Coronavirus 2[tw] OR SARS-CoV-2 Virus[tw] OR Severe Acute Respiratory Syndrome Coronavirus 2 Infection[tw] OR SARS-CoV-2[mh] OR 2019 Novel Coronavirus[tw] OR COVID-19 Virus[tw] OR COVID19 Virus[tw] OR Coronavirus Disease 2019 Virus[tw] OR Severe Acute Respiratory Syndrome Coronavirus 2[tw] OR Wuhan Coronavirus[tw] OR coronavirus[tw]) AND (Lactobacillus[mh] OR Lactobacillus[tw])

Filter: Publication data: 2020/01/01 to 2023/08/07

Search strategy to EMBASE:

('coronavirus disease 2019'/exp OR 'coronavirus disease 2019':ti,ab,kw OR ‘2019 Novel Coronavirus Disease’:ti,ab,kw OR ‘2019 Novel Coronavirus Infection’:ti,ab,kw OR ‘COVID-19 Pandemic*’:ti,ab,kw OR ‘COVID-19 Virus Disease’:ti,ab,kw OR ‘COVID-19 Virus Infection’:ti,ab,kw OR ‘Coronavirus Disease 2019’:ti,ab,kw OR ‘Coronavirus Disease-19’:ti,ab,kw OR ‘SARS Coronavirus 2 Infection’:ti,ab,kw OR ‘SARS-CoV-2 Infection’:ti,ab,kw OR ‘SARS Coronavirus 2’:ti,ab,kw OR ‘SARS-CoV-2 Virus’:ti,ab,kw OR ‘Severe Acute Respiratory Syndrome Coronavirus 2 Infection’:ti,ab,kw OR 'Severe acute respiratory syndrome coronavirus 2'/exp OR ‘2019 Novel Coronavirus’:ti,ab,kw OR ‘COVID-19 Virus’:ti,ab,kw OR ‘COVID19 Virus’:ti,ab,kw OR ‘Coronavirus Disease 2019 Virus’:ti,ab,kw OR ‘Severe Acute Respiratory Syndrome Coronavirus 2’:ti,ab,kw OR ‘Wuhan Coronavirus’:ti,ab,kw OR ‘coronavirus’:ti,ab,kw) AND (Lactobacillus/exp OR Lactobacillus:ti,ab,kw)

Filter: Years: 2020-2023

Search strategy to Scopus:

TITLE-ABS(“coronavirus disease 2019” OR “2019 Novel Coronavirus Disease” OR “2019 Novel Coronavirus Infection” OR “COVID-19 Pandemic*” OR “COVID-19 Virus Disease” OR “COVID-19 Virus Infection” OR “Coronavirus Disease 2019” OR “Coronavirus Disease-19” OR ”SARS Coronavirus 2 Infection” OR “SARS-CoV-2 Infection” OR “SARS Coronavirus 2” OR “SARS-CoV-2 Virus” OR “Severe Acute Respiratory Syndrome Coronavirus 2 Infection” OR “Severe acute respiratory syndrome coronavirus 2” OR “2019 Novel Coronavirus” OR “COVID-19 Virus” OR “COVID19 Virus” OR “Coronavirus Disease 2019 Virus” OR “Severe Acute Respiratory Syndrome Coronavirus 2” OR “Wuhan Coronavirus” OR coronavirus) AND TITLE-ABS(Lactobacillus)

Filter: Years: 2019 to 2023

Search strategy to Web of Science:

(COVID-19 OR 2019 Novel Coronavirus Disease OR 2019 Novel Coronavirus Infection OR COVID-19 Pandemic* OR COVID-19 Virus Disease OR COVID-19 Virus Infection OR Coronavirus Disease 2019 OR Coronavirus Disease-19 OR SARS Coronavirus 2 Infection OR SARS-CoV-2 Infection OR SARS Coronavirus 2 OR SARS-CoV-2 Virus OR Severe Acute Respiratory Syndrome Coronavirus 2 Infection OR SARS-CoV-2 OR 2019 Novel Coronavirus OR COVID-19 Virus OR COVID19 Virus OR Coronavirus Disease 2019 Virus OR Severe Acute Respiratory Syndrome Coronavirus 2 OR Wuhan Coronavirus OR coronavirus) AND (Lactobacillus)

Publication data: 2020, 2021, 2022, 2023

Quality assessment - Joanna Briggs Institute’s critical appraisal tools

Cross-sectional										
Study	Q1	Q2	Q3	Q4	Q5	Q6	Q7	Q8	Total	Quality
Jiang, Z., et al.; 2023	U	N	Y	Y	N	NA	Y	Y	4	Moderate
Soffritti, I., et al.; 2021	Y	Y	Y	Y	Y	U	Y	Y	7	High

Note: It was categorized as “Low” when the study obtained up to three “Yes” answers to the evaluated items; “Moderate” when the study obtained five or six “Yes” answers; and “High” when the study reached seven or more “Yes” answers.

Case-control												
Study	Q1	Q2	Q3	Q4	Q5	Q6	Q7	Q8	Q9	Q10	Total	Quality
Gaibani, P., et al.; 2021	Y	N	U	Y	NA	N	NA	Y	Y	Y	5	Moderate
Maeda, Y.: et al.; 2022	Y	Y	U	Y	U	N	NA	Y	Y	Y	6	Moderate
Wu, Y..J., et al.; 2021	Y	U	U	Y	U	N	NA	Y	Y	Y	5	Moderate
Yeoh, Y.K. et al.	Y	Y	U	Y	U	Y	Y	Y	Y	Y	8	High

Note: It was categorized as "Low" when the study obtained up to 4 "Yes" answers for the evaluated items; "Moderate" when the study obtained 5-7 "Yes" answers; and "High" when the study reached 8 or more "Yes" answers.

Cohort													
Study	Q1	Q2	Q3	Q4	Q5	Q6	Q7	Q8	Q9	Q10	Q11	Total	Quality
Kim, J.G., et al.; 2023	Y	Y	Y	Y	Y	NA	Y	Y	Y	NA	Y	9	High
Rosas-Salazar,C., et al., 2023	Y	Y	Y	N	NA	NA	Y	Y	Y	NA	Y	7	Moderate
Suskun, C. et al.	Y	Y	Y	N	NA	NA	Y	Y	Y	NA	Y	7	Moderate

Note: It was categorized as "Low" when the study obtained up to 4 "Yes" answers for the evaluated items; "Moderate" when the study obtained 5-8 "Yes" answers; and "High" when the study reached 9 or more "Yes" answers.
